# Blended learning in undergraduate dental education: a global pilot study

**DOI:** 10.1080/10872981.2023.2171700

**Published:** 2023-02-07

**Authors:** Kamran Ali, E. S. A. Alhaija, Mahwish Raja, Daniel Zahra, Zoe L Brookes, Ewen McColl, Sobia Zafar, Barbara Kirnbauer, Ahed M. Al Wahadni, Rami S. Al-Fodeh, Thikrayat Ghazi Bani-Hani, Saba O Daher, Hasan O Daher

**Affiliations:** a Qatar University, QU Health College of Dental Medicine, Doha, Qatar; b School of Psychology, University of Plymouth University, Plymouth, UK; c Paediatric Dentistry, Queensland University, Brisbane, Australia; d Department of Oral and Maxillofacial Surgery, Graz University, Graz, Austria; e College of Dentistry and Medicine, Jordan University of Science and Technology, Irbid, Jordan

**Keywords:** Dental, blended learning, students, online, undergraduate education

## Abstract

**Aims:**

To explore the global trends in blended learning in undergraduate dental education during the COVID pandemic and during the recovery phase by engaging with the students and faculty and evaluate the implications for dental education in the post-COVID era.

**Methods:**

It was a pilot cross-sectional study which employed a convenience sampling technique to recruit representatives of dental faculty and undergraduate students in 80 dental institutions globally. A previously validated questionnaire consisting of a combination of closed and open-ended items was used for data collection. Responses to these online questionnaires were processed and analysed using the R statistical computing environment.

**Results:**

A total of 320 dental students and 169 faculty members from 47 different dental institutions participated in the study. Video and Live Online Tutorials were considered to be the most effective method of online learning followed by online question banks by both groups. Significant differences were noted between faculty and students regarding time spent and effectiveness of online teaching and learning, respectively, both before and after the start of COVID. The results highlight the faculty need to engage more closely with the students to address their learning needs. Finally, the participants provided several recommendations regarding the future development of teaching and learning strategies as well as assessments in the post-pandemic era.

**Conclusions:**

This is the first study which explores blended learning in dental education with participants from multiple institutions in different regions of the globe. Compared to the faculty, students considered online learning to be less interactive and preferred learning activities and all assessments to be delivered face-to-face. The results underscore the need to adapt teaching practices to suit the learning needs of the students.

## Introduction

Blended learning refers to a combination of face-to-face teaching in a classroom and online teaching and integrates traditional and e-learning [1]. Blended learning offers a more flexible approach for the learners and promotes self-regulated learning by enhancing student autonomy and motivation [2,3]. It encourages the students to develop a deeper understanding of concepts, application of knowledge, problem-solving, and clinical reasoning skills [2,4–6].

With the availability and increasing use of the internet, e-learning has become increasingly popular in medical education allowing learning to transcend the boundaries of time and space and enhances the effectiveness of individualised learning with added flexibility [7]. However, e-learning requires access to the internet, availability of suitable platforms to deliver the learning activities, training of users, along with the need for appropriate electronic devices to participate in these learning activities [8].

Although blended learning approaches have been used in healthcare education for nearly two decades, an unprecedented increase in the use of e-learning has been witnessed globally since the start of the COVID-19 pandemic. A switch to e-learning was largely mandated by social distancing and cross-infection control measures required by the governments across the world to control the spread of COVID. Notwithstanding some challenges and barriers to e-learning, experiences during the pandemic have highlighted numerous benefits of e-learning for the stakeholders. Given the global trends, it is safe to say that blended learning approaches are likely to be used more frequently and on a much larger scale in contemporary healthcare education [9,10]. A plethora of literature has been published on the impact of COVID-19 on healthcare education since the start of the pandemic. However, most studies explore the experiences of stakeholders from individual institutions and/or countries and involve a single stakeholder group, i.e., students or faculty. The aim of this study was to explore the perceptions and experiences of both the students and faculty using the same research instrument and gain a snapshot of the global trends in the online teaching and learning in undergraduate dental education before and after the start of the COVID-19 pandemic and evaluate implications for the post-pandemic era.

### Conceptual framework

The conceptual framework of this study was underpinned by Biggs’ principle of constructive alignment which is built on the constructivist learning theory [11]. It asserts that learning occurs through the construction of knowledge, based on a combination of personal experiences, and social interactions. Alignment of learning outcomes, teaching/learning activities, and assessments offers additional educational benefits [12]. Higher education involves a situational learning approach, to develop self-directed learners who are able to adapt to the learning environments [13]. The COVID-19 pandemic mandated several modifications in the teaching and learning with an increasing reliance on online delivery of education to mitigate against the social distancing requirements. As a result, a blended learning approach was adopted by dental institutions globally with an increasing need for self-directed learning by students using diverse resources. This approach is particularly important to healthcare professionals including dentists who must demonstrate self-directed learning to keep their knowledge and skills updated for the duration of their professional career [14,15].

## Methods

*Ethical considerations*: Ethical approval was obtained from the Institutional Review Board Qatar University (Reference number: QU-IRB 1614-E/21) and the Research Ethics and Integrity Committee, The University of Queensland, Australia (Approval No. 2021/HE002445).

*Study design and study setting*: The study design was a cross-sectional online survey, which was carried out as *a* global pilot study, and representatives of dental faculty staff and undergraduate dental students in 80 dental institutions were invited to participate in an online survey.

*Sampling technique and participants*: A convenience sampling technique was used to recruit dental faculty and undergraduate students. Invites to participate in the research were sent by email to potential participants using professional channels in the prospective institutions. The invites were accompanied by a participant information sheet explaining the purpose and scope of the study.

*Research instrument*: A previously validated questionnaire aimed at exploring the experiences of online learning, and perceived benefits and barriers to online learning were used for data collection [16]. The survey inventory was based on 20 items consisting of a combination of closed and open-ended questions. Separate versions were created for the Students (APPENDIX 1) and Faculty (APPENDIX 2) to explore their experiences of online teaching and learning, respectively.

*Data collection*: The participants were invited to complete an online questionnaire using Google Forms. Prior to accessing the questionnaire, each participant was required to sign a mandatory electronic consent form to confirm they understood the purpose of the study; their participation was voluntary; and that the data was processed anonymously. The participants were also asked to confirm that this was the first time they were providing their responses to prevent multiple responses. Data were collected from 1 December 2021 to 1 February 2022. Reminders were sent after 2 weeks of the initial invites.

*Data analysis*: Responses to these online questionnaires were processed and analysed using the R statistical computing environment. Where response options were descriptive, frequency-based summaries have been presented. Where response options were categorical and compared across multiple factors, chi-squared analyses have been used where appropriate. Where cross-tabulation of two factors resulted in small numbers of individuals in some subgroups, *p*-values were computed by Monte Carlo simulation with 10,000 replicates. However, unless these differed from the results of the unadjusted analyses, the unadjusted values are reported for simplicity. Where responses provide continuous values, or groups are compared on a continuous scale, *t*-tests have been used to compare group means or change.

## Results

A total of 320 student responses and 169 faculty responses were provided from 47 different dental institutions based in Asia, Europe, Africa, Australia, North America, and Canada.

### Student sample summary

*Gender*: Of the 320 student responses, 218 (68.13%) reported their Gender as Female, 98 (30.63%) as Male. The remainder preferred not to report their Gender (*n* = 2, 0.63%), or did not respond to the item (*n* = 2, 0.63%).

*Stage of study*: Responses covered students at all stages (year) of study (Stage 1, *n* = 43, 13.34%; Stage 2, *n* = 54, 16.88%; Stage 3, *n* = 36, 11.25%; Stage 4, *n* = 58, 18.13%; Stage 5, *n* = 104, 32.50%; Stage 6, *n* = 3, 0.94%;), as well as some undertaking internships (*n* = 16, 5.00%;), or reporting having graduated (*n* = 3, 0.95%). Three respondents did not answer this item (0.94%).

*Location*: The majority of student respondents reported their location as being in Asia (*n* = 275, 85.94%), followed by minorities from Europe (*n* = 23, 7.19%), Africa (*n* = 13, 4.06%), Australia (*n* = 3, 0.94%), and South America (*n* = 1, 0.31%), with 5 (1.56%) not providing a response.

### Faculty sample summary

*Gender*: Of the 169 responses from faculty members, 74 (43.79%) reported their Gender as Female, 93 (55.03%) as Male, with one (0.59%) preferring not to say, and one (0.59%) not providing the information.

*Location*: The majority of faculty respondents reported their location as being in Asia (*n* = 135, 79.88%), followed by minorities from Africa (*n* = 12, 7.10%), Europe (*n* = 9, 5.33%), Australia (*n* = 7, 4.14%), and North America (*n* = 4, 2.37%), with 2 (1.18%) not providing a response.

*Teaching experience*: Teaching experience among the faculty respondents varied between 1 and 5 years (*n* = 31, 18.34%), 6–0 years (*n* = 39, 23.08%), 11–5 years (*n* = 45, 26.63%), 16–0 years (*n* = 24, 14.20%), and 28 (16.57%) reporting 20+ years of teaching experience. Two did not provide this information (1.18%).

*Role*: The majority of respondents indicated they were clinical teachers (*n* = 103, 60.95%), with small numbers of respondents also indicating they had research roles, taught basic sciences, and/or held some supervisory and lecturing roles, mostly alongside clinical work.

*Online learning experiences pre-COVID-19*: Responses to the question ‘Prior to COVID-19 pandemic, which online learning platforms/resources did you use in your teaching/learning?’ show a statistically significant difference in platform usage between Faculty and Student responses (*Χ*^2^_(5, *n* = 820)_ = 87.89, *p* < 0.001; ‘Missing’ responses excluded). Details are shown in Table 1. Responses from year 1 students have been excluded for questions related to pre-COVID learning as they would not have experienced the pre-COVID learning environment in their dental school.

**Table 1.** Pre-COVID 19 platform usage by faculty and students.

	Faculty		Students			
Platform	n	%*	n	%*	X^2^	*p*
Live Online Tutorials (Internal)	47	27.81	119	39.19	9.67	0.002
Live Online Tutorials (External)	11	6.51	37	11.56	4.44	0.035
Video Tutorials**	76	44.97	217	67.81	50.39	<0.001
Online Banks	32	18.93	86	26.88	7.30	0.007
Digital Flashcards	5	2.96	59	18.44	27.26	<0.001
None	47	27.81	15	4.69	42.13	<0.001
Missing	2	1.18	2	0.25	—	—

*Percentage of total Faculty/Student respondents overall, not responses to this item.

**Pre-recorded tutorials such as those available on YouTube and Osmosis, whereas Live Online Tutorials refer to those delivered in real-time via platforms such as Zoom.

*Perceived effectiveness of online learning methods*: The average effectiveness rankings for each method of learning as reported by Faculty and Student respondents are given in Table 2. For each method, the sum of the ranks given across all respondents was averaged: Most Effective = 1, Least Effective = 5.

**Table 2.** Mean effectiveness rank for learning methods.

Students		Faculty	
Resource	Mean Rank	Resource	Mean Rank
Video Tutorials	1.92	Live Online Tutorials (Internal)	1.88
Live Online Tutorials (Internal)	2.09	Video Tutorials	2.39
Live Online Tutorials (External)	3.18	Live Online Tutorials (External)	2.49
Online Question Banks	3.37	Online Question Banks	3.44
Digital Flashcards	4.14	Digital Flashcards	3.92

**Figure 1.**
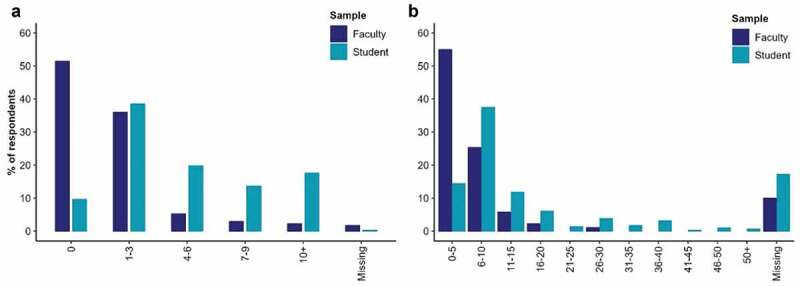
Hours spent on online learning a) Pre-COVID-19. b) After the outbreak COVID-19.

*Time spent on online learning, pre- and after the start of COVID*: The number of hours that each respondent reports spending online prior to the outbreak of the COVID-19 pandemic, by Faculty (Teaching) and Students (Learning), is shown in Figure 1a. These distributions suggest statistically significant variation in use of online platforms before the pandemic between Faculty and Students, with Students spending far more hours online (Χ2(4, *n* = 485) = 134.98, *n* < 0.001; ‘Missing’ responses excluded)

The question on how long respondents spent on online learning during the COVID-19 pandemic was open ended and received a wide variety of responses and formats. These have been reduced to numeric values and categorised into 5-hour intervals and summarised in Figure 1b. These distributions suggest statistically significant variation in the use of online learning after the outbreak of the pandemic between Faculty and Students, with Students spending more hours online (*Χ*^2^_(10, *n* = 381)_ = 84.92, *p* < 0.001; ‘Missing’ responses excluded); Faculty averaging 5.49 (*SD* = 4.73) hours, Students averaging 13.72 (*SD* = 12.39) hours (*t*(315.99)=-9.09, *p* < 0.001).

*Institutional adaptations*: Adaptations to online teaching and learning in response to COVID-19 reported by Faculty and Students are shown in Table 3. The reported adaptations differ between Faculty and Students (*Χ*^2^_(10, *n* = 952)_ = 29.34, *p* < 0.001).

**Table 3.** Adaptations in response to the COVID-19 pandemic.

	Faculty		Students			
Adaptation	n	%*	n	%*	X^2^	p
New online learning platform with new resources	66	39.05	113	35.09	0.59	0.443
New resources on an existing platform	93	55.03	111	34.47	18.45	<0.001
Live tutorials via Zoom or similar platforms	84	49.70	251	77.95	39.50	<0.001
Pre-recorded lectures/tutorials	63	37.28	171	53.11	10.51	0.001

* *Percentage of total Faculty/Student respondents overall, not responses to this item*.

*Interactions during online sessions*: The interactivity ratings for Online Sessions were positively rated by 40.2% of Faculty, with majority indicated as interactive (33.1%), and 22.5% indicated as not interactive. With regard to Students 25.6% positively rated Online Sessions interactivity, with 39.4% found majority of sessions as interactive, and 27.1% did not find them interactive. When asked to indicate what made these sessions interactive, Speech was highly reported by Faculty (80%) and Students (70%); this was followed by Chatbox with 45% of Faculty, and 52.8% of Students; and Quiz was reported as interactive by 26.6% by Faculty and 32.2% of Students.

*Content of online learning sessions*: Among Faculty respondents, 45.56% (*n* = 77) reported that the content of their online teaching was determined by university policy, 24.26% (*n* = 41) by government regulations, 22.49% (*n* = 38) by a pre-set curriculum, and 6.51% (*n* = 11) by student requests. The remaining 1.18% (*n* = 2) did not provide a response. Among Student respondents, 60.63% (*n* = 194) reported that their online learning followed a pre-set curriculum, 7.50% (*n* = 24) reported it followed student requests, and 29.38% (*n* = 94) reported that both pre-set curricula and student-requests directed their online learning. The remaining 2.50% (*n* = 8) did not provide a response.

**Figure 2.**
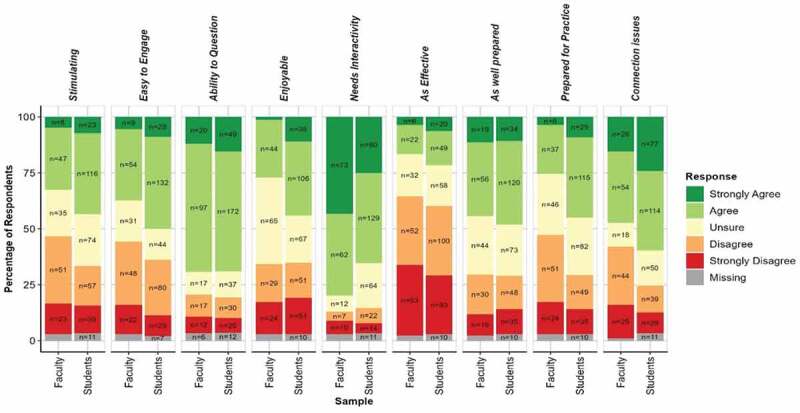
Perceptions of Online teaching and learning.

*Perceptions of online teaching and learning*: Faculty and Students were asked to rate their agreement (coded as Strongly Agree = 2 to Strongly Disagree = −2) with the extent to which they felt that online teaching and learning was stimulating (t = −2.64; p = 0.009), easy to engage with (t = −2.49; p = 0.013, provided a context in which questions could be asked (t = −0.58; p = 0.56), was enjoyable (t = −2.33; p = 0.02), needed to be more interactive (t = 3.13; p = 0.002), was as effective as face-to-face sessions (t = −1.59; p = 0.112), was delivered by well-prepared staff (t = −0.28; p = 0.777), prepared students well for their professions (t = −4.47; *p* = <0.001), and could be impacted by problematic internet connections (t = −3.82; *p* = <0.001). The percentage of agreement with each statement/dimension is shown in Figure 2.

*Enjoyable aspects of online teaching and learning*: Respondents were asked to select all aspects of online teaching and learning that they enjoyed, choosing from the lack of travel, cost savings, interactivity, ability to ask questions, comfort, ability to learn at their own pace, and the flexibility (Table 4). The perceived barriers to online teaching and learning indicated by respondents indicated are given in Table 4.

**Table 4.** Enjoyable aspects and perceived barriers to online teaching and learning.

	Faculty		Students			*X*^*2*^	*p*
	*n*	*%*	*n*	*%*			
**Enjoyable Aspects of online teaching and learning**							
No Travel	95	56.21	187		58.07	0.09	0.764
Cost Saving	67	39.64	158		49.07	3.59	0.058
Interactive	44	26.04	67		20.81	1.44	0.229
Questions	41	24.26	94		29.19	1.12	0.291
Comfortable	81	47.93	207		64.29	11.56	<0.001
Pace	80	47.34	187		58.07	4.73	0.030
Flexibility	116	68.64	203		63.04	1.28	0.256
**Perceived barriers of online teaching and learning**							
Internet connectivity	123	72.78	245		76.09	0.48	0.49
Timing	48	28.40	135		41.93	8.10	0.004
Family distractions	88	52.07	191		59.32	2.086	0.149
Lack of suitable space	33	19.53	89		27.64	3.48	0.062
Unavailability of devices	33.73		65		20.19	10.17	0.001

*Online teaching as a substitute to clinical teaching and direct patient contact*: When asked whether they thought online teaching had replaced clinical teaching experienced with direct patient contact, most respondents, both Faculty (*n* = 139, 82.25%) and Students (*n* = 221, 68.63%), said ‘no’, some said ‘yes’ in both groups; Faculty (*n* = 10, 5.92%) and Students (*n* = 46, 14.29%), and some said ‘to some extent’; Faculty (*n* = 20, 11.83%) and Students (*n* = 52, 16.15%). Three (0.93%) student respondents did not answer. These proportions vary between faculty and Students (*Χ*^2^_(2, *n* = 491)_ = 10.97, *p* = 0.004).

*Online teaching and learning of practical dental skills*: When asked whether they thought clinical skills could be taught online, the vast majority of both groups said no, though significantly more students than faculty said yes (*Χ*^2^_(2, *n* = 486)_ = 14.35, *p* < 0.001; missing group excluded).

*Impact of COVID-19 on assessments*: When asked if COVID-19 had affected their assessments, most Faculty and Students said yes (*n* = 124, 73.37% Faculty: *n* = 190, 59.01% Students – Excluding those in Year 1). The distribution of Yes, No, and NA between Students and faculty differed significantly (*Χ*^2^_(2, *n* = 445)_ = 10.52, *p* = 0.005; missing group excluded), with larger proportions of Students reporting no impact or not applicable. The specific areas which respondents thought were impacted by COVID-19 are shown in Table 5. A summary of the plans for and current administration of written and clinical assessments in the current academic year is summarised in Table 5. Responses from year 1 students have been excluded for these questions as they would not have experienced the pre-COVID assessments in their dental school.

**Table 5.** Impact of COVID-19 on written and clinical assessments.

	Faculty		Students		*X*^*2*^	*p*
	*n*	*%*	*n*	*%*		
**Impact of COVID-19 on Written Assessments**						
Remote assessment (Online)	81	47.93	149	53.79	1.22	0.270
Face-to-Face (Campus)	34	20.12	63	22.74	0.28	0.594
Written assessments postponed	16	9.47	41	14.80	2.22	0.136
Written assessment cancelled	18	10.65	26	9.39	0.07	0.787
Not Applicable	18	10.65	47	16.97	2.88	0.0.090
**Impact of COVID-19 on Clinical Assessments**						
Remote assessment (e.g., Virtual Patients)	26	15.38	48	17.33	0.16	0.686
Face-to-Face (Campus)	55	32.54	91	32.85	<0.001	>0.999
Clinical assessments postponed	37	21.89	74	26.71	1.06	0.303
Clinical assessment cancelled	25	14.79	56	20.22	1.73	0.189
Not Applicable	25	14.79	60	21.66	2.78	0.095
**Administration of Written Assessments in the current year**						
Remote assessment (Online)	16	9.88	38	13.06	1.41	0.236
Face-to-Face (Campus)	97	59.88	122	41.92	6.96	0.008
Both	35	21.60	52	17.87	0.14	0.706
Don’t Know/Unsure	14	8.64	79	27.15	24.83	<0.001
**Administration of Clinical Assessments in the current year**						
Remote assessment (e.g., Virtual Patients)	3	1.81	18	6.32	4.22	0.040
Face-to-Face (Campus)	124	74.70	168	58.95	6.96	0.008
Both	25	15.06	39	13.68	0.005	0.945
Don’t Know/Unsure	14	8.43	60	21.05	12.62	<0.001

### Qualitative data: responses to open-ended items

*Future of* blended learn*ing*: When participants were asked about the future of blended learning in dental education, over 70% of the faculty viewed blended learning as an appropriate approach to teaching and envisaged its use more frequently. Blended learning was perceived to be an effective method for didactic and knowledge-based teaching as well as student-led workshops and seminars. However, it was not considered appropriate for learning practical skills in simulated or clinical settings which require face-to-face training. Similarly, it is not possible or appropriate to conduct practical and clinical assessments in dentistry remotely and would continue to be undertaken on-site.

Mixed views were expressed regarding remote delivery of knowledge-based assessments as faculty staff considered cheating by students to be a potential risk. However, faculty members who had experienced the use of remote proctoring using online assessment platforms reported that knowledge-based assessments could continue to be administered remotely. The student participants also envisaged that blended approaches are likely to be used more frequently for knowledge-based learning activities. However, approximately 55% of student participants preferred face-to-face learning and assessments whenever possible. The students emphasized the online learning was less interactive and face-to-face interactions with their peers and faculty influenced their learning positively. Less than 25% of students were keen for knowledge-based assessments to be conducted remotely.

*Challenges of blended learning*: Faculty and students from developing countries considered the availability of uninterrupted, high-speed internet connection to be the main challenge for blended learning. Technical problems hardware and software of internet devices used during blended learning sessions were also reported to be an issue by both faculty and students. The faculty were also concerned about students being distracted during large-group online sessions and difficulties in monitoring their involvement and attentiveness. Additionally, the students reported lack of technical expertise with some faculty members when using online platforms which could result in delays, interruptions, and even cancellation of teaching activities.

## Discussion

The first case of COVID-19 was reported from China in December 2019, followed by the identification of COVID in different countries until the World Health Organization (WHO) declared COVID-19 a global pandemic on 11 March 2020 [17]. The COVID-19 pandemic has highlighted the lack of preparedness of educational providers. Nevertheless, dental educators made huge progress to mitigate against the impact of COVID-19 and swiftly adapted to using online platforms such as, Zoom, Google Rooms, Skype, Microsoft Teams, and WebEx. Additionally, numerous bespoke applications have been developed for remote teaching, assessments, meetings, and patient consultations. These measures involved a sharp learning curve for the dental educators and students to use technology efficiently in a short period of time [18].

A fundamental goal of undergraduate dental education across the globe is to produce competent dental graduates who can provide safe and effective care to the communities [15,19,20]. Dental students and faculty at dental institutions in different countries were invited to gain a view of the bigger picture to identify the commonalities in the perceptions and experiences regarding multiple dimensions of online teaching, learning, and assessments before and after the start of COVID-19 pandemic. Significant differences in the responses were observed between faculty and students for several items on the questionnaire including the type of online resources utilized; time spent online (pre- and post-COVID-19); perceived effectiveness of online activities; and opportunities of interactions during online activities.

Students spent more time on online learning both before and after the start of COVID compared to the online teaching by the faculty, reflecting the extra time spent on self-directed learning activities. Video Tutorials and Live Online Tutorials were reported to be the most effective method of learning by the students and faculty alike. However, students appeared to utilise video tutorials more frequently. Faculty used video tutorials less frequently in their teaching and this may be related to the time required to prepare video tutorials. These observations may also imply that students use external video resources in their learning in addition to ones provided by the faculty at their own institution(s).

Despite spending more time on exploring online learning resources, nearly 75% student participants did not consider online learning to be sufficiently interactive and preferred face-to-face learning activities. This was in contrast to the views of over 40% of faculty participants who endorsed online teaching and learning. Significant differences in the perspectives of the students and the faculty underscore the need to develop a better understanding of the learning needs and resources preferred by the students. Students are the major stakeholders in dental education, and it would be inappropriate to view students merely as recipients. Transformation of students into independent learners can be best achieved by recognizing them as ‘partners’ in education [21–23]. This approach entails developing a better understanding of the learning needs of students through representation and active participation of students in curriculum development, teaching and learning activities and assessments. Faculty need to work closely with students to understand learning resources preferred by the students and adapt their teaching to support student learning rather than focusing solely on the delivery of teaching content.

Online digital assessment platforms have been in use for several years and are deemed suitable for knowledge-based assessments. They allow secure storage of assessment content, design, and blueprinting of assessments based on a range of formats with a full audit trail of item usage, along with performance of items as well as candidates. Moreover, contemporary assessment platforms also offer options for online and offline delivery of assessments on campus as well as the option of remote proctoring. The latter option has been particularly beneficial during the peak of COVID-19 pandemic which necessitated social distancing measures making on-site delivery of assessments challenging [24]. It is recognized that commercially available online assessment platforms entail significant costs and investment in human resources. Commercial providers routinely charge a setup fee with additional expenses for maintaining the commercial services. Also, effective use of online assessment platforms requires administrative support and staff training. It is not surprising that dental institutions with financial limitations may not be able to invest in such resources. A common concern expressed by the faculty with remote online delivery of assessments is the risk of cheating by the students [25]. Faculty participants in this study also expressed similar concerns. However, less than 25% of student participants preferred remote delivery of knowledge-based assessments. Therefore, knowledge-based assessments should preferably be delivered on campus.

Administration of clinical assessments in dentistry requires direct patient contact, making remote assessment impractical. Nevertheless, numerous medical and dental institutions managed to deliver objective structured clinical examinations (OSCEs) virtually or using a hybrid approach during the peak of COVID-19 pandemic [26–30]. Although virtual and simulated examinations have been advocated to assess students’ competency in a range of attributes, such approaches do not directly assess the same facets competency as examinations conducted face-to-face [31]. The findings of this study further highlight the limitations of virtual OSCEs as perceived by the stakeholders. Both student and faculty participants did not consider virtual clinical assessments to be a replacement for face-to-face clinical assessments involving interactions with real patients. The results of the present study also underscore the challenges of clinical examinations aimed at assessing students’ competence in performing clinical dental procedures.

A few limitations of this study need to be acknowledged. Firstly, dental academics in several institutions across the globe were invited to participate in this study. The selection of potential institutions was based on the availability of professional contacts of the research team. Responses were achieved from approximately 59% of invited institutions. Lower response rates were achieved from Dental Schools in the US and Australia. Some participants did not provide the information related to their institution and only indicated their continental location. The item related to the institution was optional due to ethical constraints as some participants may not wish to provide this information. Given the missing information regarding institutions/countries of some participants, it was not possible to analyze the impact of participants’ institution/location on their perspectives regarding online teaching and learning. Nevertheless, it was a pilot study and it does provide a snapshot of the global picture to gauge the experiences of stakeholders in dental education. In any case, the findings may have limited generalizability and should be interpreted with a degree of caution.

Blended learning is well established in dental education, and dental educators must remain prepared for remote assessments if required in the future. Future large-scale studies aimed at gauging global trends in dental education may be best undertaken with support from professional organizations such as the Association for Dental Education in Europe (ADEE), the American Dental Education Association (ADEA), and similar organisations in other parts of the world. Such initiatives would require creation and maintenance of a dedicated database to engage with dental institutions worldwide.

## Conclusion

This is the first study which explores blended learning in dental education with participants from multiple institutions in different regions of the globe. It provides useful insights into the perceptions and experiences of participants representing dental students and faculty before and after the COVID-19 pandemic. Compared to the faculty, the students spent more hours on online learning. Regarding opportunities for interactions, Online Sessions were rated positively by 40.2% of Faculty, while only 25.6% Students rated them positively. Avoiding the need for travel and cost saving were considered the main advantages of online learning by participants across the board, while issues with internet connectivity was the main barrier. While online teaching and learning was considered to be effective for didactic teaching, participants did not view it as a suitable option for learning clinical and practical skills. Significant disruptions to assessments were reported especially for clinical and practical assessments by a large number of participants among both the faculty and students. The participants did not consider virtual assessments as a suitable replacement for face-to-face assessment of clinical operative skills of dental students. The participants also provided recommendations to improve blended learning approaches in dental education. The results also underscore the need to adapt teaching practices to suit the learning needs of the students. Further studies involving a larger sample with proportional representation of dental institutions from across the globe are required to ensure generalizability of the findings.

## Supplementary Material

Supplemental Material

## Data Availability

Detailed survey data are available from the corresponding author upon request.
