# Supplementary material for: Blended learning in undergraduate dental education: a global pilot study
Source: Med Educ Online. 2023 Feb 7;28(1):2171700. doi: 10.1080/10872981.2023.2171700 (PMC9930845; doi:10.1080/10872981.2023.2171700)
Supplement: Supplemental Material [file ZMEO_A_2171700_SM8396.zip › Supplementary/Appendix 2 Faculty Perceptions and Experiences of Blended Learning.pdf]

Faculty Perceptions and Experiences of Blended Learning

I Demographic Information

1. Teaching Experience

Mark only one oval.

- 1-5 Years
- 6-10 Years
- 11-15 years
- 16-20 years
- More than 20 years

2. What is your gender?

Mark only one oval.

- Female
- Male
- Prefer not to say

3. Which one of the following best describes your role

Mark only one oval.

- Teacher - Basic sciences
- Teacher - Clinical
- Researcher
- Other: ____________________

4. Location

Mark only one oval.

- Africa
- Antarctica
- Asia
- Australia
- Europe
- North America
- South America

5. Institution

6. Department

Online Teaching Experiences

1. Prior to COVID-19 pandemic, which online learning platforms/ resources did you use in your teaching?(Select all which apply)

Check all that apply.

- [ ] Video tutorials e.g. YouTube/ Osmosis
- [ ] Live tutorials via Zoom or similar platforms by your institutions
- [ ] Live tutorials via Zoom or similar platforms (external sources)
- [ ] Online question banks
- [ ] Online /digital flashcards e.g. Brainscpae, Anki
- [ ] None
- [ ] Other

2. Which method of online learning do you find to be the most effective for your students (Please rank each option of the following options from 1-5; 1=least effective, 5=most effective)

Mark only one oval per row.

	Video tutorials e.g. YouTube/ Osmosis	Live tutorials via Zoom or similar platforms by your institutions	Live tutorials via Zoom or similar platforms (external sources)	Online question banks	Online /digital flashcards e.g. Brainscpae, Anki	None
First choice	⬭	⬭	⬭	⬭	⬭	⬭
Second choice	⬭	⬭	⬭	⬭	⬭	⬭
Third choice	⬭	⬭	⬭	⬭	⬭	⬭
Fourth choice	⬭	⬭	⬭	⬭	⬭	⬭
Fifth choice	⬭	⬭	⬭	⬭	⬭	⬭

3. Prior to COVID-19 pandemic, how many hours on average did you spend on online teaching per week

Mark only one oval.

- ⬭ 0
- ⬭ 1-3
- ⬭ 4-6
- ⬭ 7-9
- ⬭ 10 or more

4. During the COVID-19 pandemic, how has your institution adapted to online teaching and learning?

Mark only one oval.

- Introduced a new online learning platform with new resources
- Introduced new resources on an existing platform
- Delivered live tutorials via Zoom or similar platforms
- Delivered pre-recorded lectures/ tutorials
- Other: ______________________

5. Are your online teaching sessions interactive?

Mark only one oval.

- Yes
- No
- Majority are interactive
- Majority are not interactive

6. What makes your online teaching sessions interactive?

Mark only one oval.

- Opportunity to interact via chatbox
- Opportunity to interact via speech
- Live quiz
- Other (please specify)

7. Does your online teaching follow a pre-set curriculum or is it based on student requests?

Mark only one oval.

- Pre-set curriculum
- Student requests
- Both

8. During the current COVID-19 pandemic, how many hours of online teaching do you provide per week?

____

9. Please respond to the following statements in the context of online teaching

Mark only one oval per row.

	Strongly disagree	Disagree	Unsure	Agree	Strongly agree
Online teaching iis stimulating for the students					
The students find iitt easy to engage with the session					
The students are able to ask questions					
The students enjoy online learning					
Online teaching needs tto be more interactive					
Online teaching iis as effective as face--to--face teaching					
The teachers are well-prepared for online teaching sessions					
The students are being prepared well for their profession					
My internet connection can be problematic					

10. Which aspects of online teaching do you enjoy (Please select all which apply)

Mark only one oval.

- No travel
- Cost saving
- Interactive
- Ability to ask questions
- More comfortable
- Ability to learn at own pace
- Flexibility
- Other: ____________________

11. What do you feel are the barriers to online do you enjoy (Please select all which apply)

Mark only one oval.

- Internet connection
- Timing of tutorials
- Family distractions
- Lack of space
- Lack of devices
- Other: ____________________

12. Do you think online teaching has replaced clinical teaching experienced with direct patient contact?

Mark only one oval.

- Yes
- No
- To some extent

13. Do you feel students can learn practical skills through online teaching?

Mark only one oval.

- Yes
- No
- To some extent

14. Have your assessments been affected by COVID-19?

Mark only one oval.

- Yes
- No
- N/A

15.How did COVID-19 affect your written assessments in the last academic year?

Mark only one oval.

- Remote assessments (online)
- Face-to-face assessments (on campus)
- Written assessments postponed
- Written assessments cancelled
- N/A

16. How did COVID-19 affect your clinical assessments in the last academic year?

Mark only one oval.

- Remote assessments (e.g., using virtual patients)
- Face-to-face assessments (on campus)
- Clinical assessments postponed
- Clinical assessments cancelled
- N/A

17. How do you plan to administer written assessments in the current academic year?

Mark only one oval.

- Remote assessments (online)
- Face-to-face assessments (on campus)
- Written assessments postponed
- Written assessments cancelled
- N/A

18. How do you plan to administer clinical assessments in the current academic year?

Mark only one oval.

- Remote assessments (e.g., virtual patients)
- Face-to-face assessments (on campus)
- Clinical assessments postponed
- Clinical assessments cancelled
- N/A

Open-ended Questions

19. In your opinion, what is the future of blended learning in dental education?

20. What is the main challenge of blended learning in dental education at your institution?
